# Editorial: Fuzzy Interactions: Many Facets of Protein Binding

**DOI:** 10.3389/fmolb.2022.947215

**Published:** 2022-06-20

**Authors:** Damiano Piovesan, Miguel Arbesú, Monika Fuxreiter, Miquel Pons

**Affiliations:** ^1^ Department of Biomedical Sciences, University of Padova, Padova, Italy; ^2^ Department of NMR-Supported Structural Biology, Leibniz-Forschungsinstitut für Molekulare Pharmakologie, Berlin, Germany; ^3^ Biomolecular NMR Lab, Department of Inorganic and Organic Chemistry, Universitat de Barcelona (UB), Barcelona, Spain

**Keywords:** intrinsically disordered proteins, fuzzy complexes, disordered drug targets, amyloids, chaperones, transcription regulation, conformational ensembles, thermodynamics

The complexity of cellular processes is enabled by the adaptability of protein interactions. This includes binding of the same protein to different substrates or fine-tuning regulation of the assemblies according to the cellular conditions. Protein complexes involving intrinsically disordered proteins (IDPs) are characterized by an ensemble of heterogeneous conformations, which often exhibit a conformational exchange in the bound form (Arbesu et al., 2017). This enables adaptability, as the populations of conformational sub-states can be altered by the cellular conditions (Gianni et al., 2021). Fuzzy complexes exhibit context-dependent binding modes involving proteins with a wide-range of structural order: a continuum ranging from rather rigid polymorphic conformations to highly dynamic complexes. Fuzzy interactions in stochastic assemblies and higher-order structures lead to changes in binding modes according to cellular signals and milieu resulting in regulated formation and function (Wu and Fuxreiter, 2016).

The complex interaction behavior of proteins forming fuzzy complexes is far from being understood. It is challenging for most traditional structure-determination techniques due to the multiplicity of binding modes, known as multimodal binding. This phenomenon can be studied in conformational ensembles of protein assemblies, which were formed under different cellular conditions or pathways (Hatos et al., 2021). Sequence-based predictions of binding sites with different interaction behaviors are available, for example by the FuzPred method (Miskei et al., 2020), yet detailed insights into the mechanisms of context-dependent interactions should be obtained from computer simulation methods combined with experimental studies. This Research Topic provides insights into recent advances in studying fuzzy interactions.

Fuzzy interactions may lead to the formation of complexes with a wide range of thermodynamic properties from weak to ultra-high affinities. Zavrtanik et al. presents a detailed thermodynamic analysis of the toxin-antitoxin HigA2-HigB2 complex, which binds with picomolar affinity. They identify a 20-residue binding region, which is responsible for the ultra-high affinity and demonstrate that interface properties of the disordered protein complex are similar to the ordered barnase-barstar complex. Analyzing binding thermodynamics they find that such ordered binding modes can be achieved by optimizing enthalpy, whereas fuzziness is promoted by optimizing entropy of binding.

Structural polymorphism as well as dynamic conformational exchange in the bound complex modulates the binding properties and impact the functional outcomes of fuzzy complexes. Nyitray et al. discusses structural studies of S100 assemblies, which exhibit both structural features. S100 proteins are small calcium-binding proteins, which regulate MAPK pathway by promoting/inhibiting phosphorylation events, which regulate formation of downstream assemblies. The authors review experimental approaches to characterizing both types of fuzziness and its role in fine-tuned regulation of signaling pathways.

The interactions of chaperones with clients should vary with the degree of unfolding of the client proteins. Sučec et al. analyzes the interactions between small heat shock proteins and mitochondrial chaperones addressing the problem of specificity. They find that energetic frustration contributes to formation of fuzzy interaction as demonstrated in case of the Spy-Im7 complex. They show that the magnitude of frustration correlates to the variability of binding modes, which provides a regulatory mechanism for chaperone function. In addition, this may serve as a protective mechanism for exposed hydrophobic sites, which must be shielded or fold to avoid aggregation.

Along this line, Pintado-Grima et al. presents a dataset of cryptic amyloidogenic sequences, containing hydrophilic residues but able to promote aggregation. These sequences are present in more than half of IDRs and may represent protein-protein interacting regions able to participate in fuzzy interactions (Vendruscolo and Fuxreiter, 2021).

Bonucci et al. discusses the effect of the cellular environment, in particular molecular crowding in shaping interaction landscapes. Using a variety of experimental techniques, they demonstrate that crowding agents only moderately alter the conformational properties of nuclear protein 1 (NUPR1), disordered interactions of which are critical for prostate cancer.

Conformational heterogeneity of fuzzy complexes can also be caused by local structural changes. Proline cis-trans isomerization is a recently recognized factor in fuzzy interactions. Alcantara et al. studies the binding of proline-rich ArkA peptides to SH3 domains using accelerated molecular dynamics simulations. They demonstrate that multiple prolines isomerize during the microsecond timescale of the simulations, which was also corroborated by circular dichroism measurements.

Variability of binding may present a challenge for traditional drug discovery methods for a broad range of targets, such as transcription factors, receptors with disordered substrate binding loops, or signaling complexes forming higher-order structures. Su et al. review the bottlenecks of targeting fuzzy complexes by small molecules. They present a variety of approaches, which can be used to tackle the problem, such as orthosteric and allosteric mechanisms that can be applied to transcription factors.

It has been increasingly recognized that protein interactions depend on the cellular context and vary with the cellular conditions. This requires changes in the binding mode between specific partners. That is, the conformational ensembles of fuzzy complexes, the population of different sub-states are modulated by the cellular environment. The current Research Topic gives insights into how such complex regulatory mechanisms take place and discusses the biophysical forces underlying fuzziness. The articles detail the experimental and computational approaches, which are applicable to study conformational heterogeneity in the bound state and linking this structural property with biological functions.
